# The Role of Acute Phase Reactants, Including α1-Acid Glycoprotein, in Predicting Onset and Severity of Retinopathy of Prematurity

**DOI:** 10.3390/diagnostics15050571

**Published:** 2025-02-27

**Authors:** Yoko Nakazawa, Tsutomu Yasukawa, Haruo Goto, Satoru Kobayashi, Kyoko Yokoi

**Affiliations:** 1 Department of Ophthalmology, Nagoya City University West Medical Center, Nagoya 462-8505, Japan; 2 Department of Ophthalmology and Visual Science, Nagoya City University Graduate School of Medical Sciences, Nagoya 467-8601, Japan; 3 Department of Pediatrics, Nagoya City University West Medical Center, Nagoya 462-8505, Japan

**Keywords:** retinopathy of prematurity, acute phase reactants, predictive factor, discriminant probability, C-reactive protein, alfa 1 acid glycoprotein, haptoglobin

## Abstract

**Background**: Retinopathy of prematurity (ROP) is a serious disease causing blindness in childhood. Gestational age (GA) and birth weight are major factors associated with the development and progression of ROP, but postnatal systemic inflammation is also an important well-known risk factor. **Methods**: This study retrospectively analyzed the relationship between systemic inflammation and ROP severity using the corrected GA (CGA), which reflects the intrinsic immaturity of the infant, rather than days of life. Three acute phase reactants (APRs) were analyzed using discriminant probability and compared with conventional ROP prediction models: C-reactive protein, α1AG, and haptoglobin. **Results**: Alpha 1AG was the best predictor of ROP onset and progression, and could be predicted with blood samples up to 30 weeks (30 W) CGA (*p* = 0.006). Incorporation of APR into the conventional GA + body weight (BW), ROP score, and Children’s Hospital of Philadelphia (CHOP) predictive models improved the decision to treat (4–5% increase in discrimination probability) and helped determine whether treatment of ROP was necessary by CGA 30 W. **Conclusions**: Therefore, simply adding α1AG protein to the assessment is useful for predicting the need to treat ROP.

## 1. Introduction

Retinopathy of prematurity (ROP) is a disease in which the condition of the retina up to 80 days of age determines lifelong vision. Although gestational age (GA) and birth weight (BW) are the main predictors of ROP onset and severity [1,2], the pathogenesis of ROP is multifactorial [3,4,5,6,7,8].

Many reports have examined the relationship between ROP severity and serum levels of inflammatory markers that reflect systemic status using the number of days since birth [9,10,11,12]. While some studies suggest that the number of days after birth may be a better predictor of ROP severity than days post menstrual age [2], one large study found that the timing of treatment based on ROP severity is more closely associated with corrected gestational age (CGA), rather than the days of age after birth [13,14]. Therefore, we felt that when examining inflammatory biomarker values to estimate ROP severity, values derived by CGA rather than days of age values would be more appropriate. We focused on three acute phase reactants (APRs) [15,16]: C-reactive protein (CRP), α1 acid glycoprotein (α1AG) [15,16,17,18], and haptoglobin (HP) [19]. These are more biologically stable than biomarkers such as cytokines and can be obtained by adding them to a normal NICU serum collection [15]. In this study, blood samples were routinely collected in the neonatal intensive care unit from birth to 35 weeks CGA to assess the predictability of ROP progression and the need for treatment based on the levels of these three APRs.

## 2. Methods

This retrospective study was approved by the institutional review board of the Western Medical Center of Nagoya City University Graduate School of Medicine (20-04-369-23), and was performed in accordance with the principles of the Declaration of Helsinki. Informed consent was obtained from both parents of all patients using an opt-out method.

### 2.1. Patients

This study involved preterm infants who were born at Nagoya City University Western Medical Center at ≤31-week GA or with a BW ≤ 1499 g and underwent ophthalmological screening for ROP in the neonatal intensive care unit (NICU) between January 2011 and December 2019. All infants were examined by a skilled ophthalmologist (YN) under pupillary dilation with 2.5% phenylephrine hydrochloride eye drops using an indirect ophthalmoscope or a wide-angle fundus camera system (Ret Cam Envision; Natus Medical Incorporated, Middleton, WI, USA) until retinopathy activity subsided. Infants who died before 36-week CGA were excluded from analysis.

### 2.2. ROP Classification

The stage of ROP in each infant was determined according to the International Classification of Retinopathy of Prematurity, 3rd edition [20]. Eyes were classified into three groups: no ROP, mild ROP (spontaneous remission of ROP), and treated ROP. Treatment of ROP was determined according to the Randomized Early Treatment of Retinopathy of Prematurity Trial [21].

### 2.3. Laboratory Studies

Venous blood samples were obtained from all patients with ROP and analyzed for serum levels of three APRs (CRP, α1AG, and HP) using a turbidimetric immunoassay (Quick Turbo; Shino-Test Corporation, Sagamihara, Japan). The assay’s limits of detection of CRP, α1AG, and HP are 0.3 mg/dL, 20 mg/dL, and 13 mg/dL, respectively.

Data were routinely collected from immediately after birth until CGA of 35 weeks. Blood samples were obtained daily during the first week of life, at least four times per week until CGA of 30 weeks, and thereafter at least once per week or more frequently when the patient was in poor general condition (except when blood collection may have worsened the condition).

### 2.4. Predictive Instrumentation and Classification

Clinical data were collected from the patients’ electronic medical records. Several general items were also investigated to assess the overall condition of the enrolled children and to compare with other studies. At baseline, the following variables were examined: GA at birth, BW, sex, pulmonary function (use of continuous positive airway pressure up to CGA of 33 weeks, use of supplemental oxygen up to CGA of 36 weeks, and respiratory distress syndrome defined by use of a surfactant), circulatory function (patent ductus arteriosus requiring ligation, anemia requiring blood transfusion at CGA ≤ 35 weeks), and brain function (intraventricular hemorrhage and periventricular leukomalacia). We examined infant weight gain at 6 weeks of age as a postnatal growth parameter for comparison with the existing predictive ROP score [22], and we assessed daily weight gain for comparison with the CHOP model [23]. The daily rate of weight gain was calculated by dividing the weekly weight change from postnatal Days 8 to 15 by 7.

### 2.5. Data Analysis

The APRs were analyzed by selecting the highest value among those at 72 h after birth, CGA of 30 weeks, 33 weeks, and 35 weeks. For infants with a GA of 31 weeks, the values at 30-week CGA were taken as the 72 h values.

Analysis of the predictive capability of the APRs at each time point was conducted based on the following categories:

**Category 1:** Comparison between patients with and without ROP, distinguishing between no ROP and the combination of mild ROP and treated ROP;

**Category 2:** Comparison between patients with and without the need for treatment in instances of ROP, specifically comparing mild ROP with treated ROP;

**Category 3:** Comparison between patients with and without the need for treatment in all instances, distinguishing between no ROP plus mild ROP and treated ROP.

For all categories, the values of the three proteins at each time point were analyzed using multivariate-adjusted analysis with calculation of the discriminant coefficient and *p*-value. In the same way, the values of the three proteins were adjusted and summed, analyzed using multivariate-adjusted analysis with calculation of the discriminant probability coefficient, efficiency, and *p*-value. The relationships between the discriminant probability values of the APRs to the GA + BW, ROP score, and CHOP models, as well as each of the three categories, were also evaluated.

All parameters were subjected to linear discriminant analysis and multivariate analysis. Continuous variables included GA, BW, daily weight gain from postnatal days 8 to 15, weight gain at postnatal 6 weeks of age, and the maximum values of the three APRs. Other parameters were treated as categorical variables.

Discriminant analysis is multiple regression analysis with the group, with or without ROP, with or without treatment for ROP, as the objective variable and several parameters (BW, GA, APRs, etc.) as explanatory variables. In this study, linear discriminant analysis was performed using the stepwise forward selection method. Mahalanobis squared distance was applied to all parameters in the discriminant analysis. The discriminant probability equation was derived from these discriminant coefficients. Cases with values greater than or equal to zero, as calculated by the discriminant probability equation, were classified into one group, while cases with values below zero were assigned to the other group. The discriminant probability reflects the accuracy rate of the discriminant analysis based on the discriminant probability equation.

All analyses were performed using SAS software ver. 9.3 (SAS Institute, Cary, NC, USA).

## 3. Results

### 3.1. Characteristics of Infants

This study involved 290 infants, and their clinical characteristics are summarized in Table 1. Of the 290 infants, 23 (8.0%) required treatment and were thus classified into the treated ROP group, 112 (38.6%) developed ROP with spontaneous remission and were thus classified into the mild ROP group, and 155 (53.4%) did not develop ROP and were thus classified into the no ROP group.

### 3.2. Multifactor Comparison of All Groups

Each systemic background factor was compared by linear discriminant analysis (Table 1). GA, BW, continuous positive airway pressure (CPAP), history of oxygen use, respiratory distress syndrome (RDS), patent ductus arteriosus (PDA) ligation, blood transfusion, and weight gain were effective predictors of ROP onset and severity (*p* < 0.001). There was a modest but significant correlation with intraventricular hemorrhage, potentially affecting the central nervous system (*p* = 0.015), whereas periventricular leukomalacia showed no relationship with the onset and severity of ROP (*p* = 0.56). Male infants were more prevalent in the treated ROP group (*p* = 0.041) (Table 1).

### 3.3. Predictability of Each APR in Categories 1, 2, and 3

CRP was significant only at a CGA of 30 weeks in Category 2 (*p* = 0.047). A1AG was highly significant at CGA of 30, 33, and 35 weeks in Categories 1, 2, and 3 (*p* < 0.01). HP was significant up to 72 h after birth and CGA of 35 weeks in Category 1 (*p* = 0.037 and 0.046, respectively) (Table 2).

### 3.4. Multivariate-Adjusted Analysis for All APRs (CRP + α1AG + HP) in Categories 1, 2, and 3

A multivariate-adjusted analysis was performed for all APRs to determine the probabilities of ROP occurrence (Category 1), ROP progression requiring treatment (Category 2), and ROP with treatment (Category 3). There were no significant differences between the groups in any categories at 72 h after birth, whereas each APR at CGA of 30, 33, and 35 weeks showed significant differences in all categories (*p* < 0.0001) (Table 3). In Category 1, the discriminant probability suggesting predictability of ROP occurrence was 66–67%. The analysis in Category 2 showed a discriminant probability of 67%, suggesting predictability of ROP progression requiring treatment. In Category 3, the discrimination probability for prediction of eyes with ROP treatment was 75–76%.

### 3.5. Predictability of GA + BW, ROP Score, and CHOP Models and Impacts of APRs (CRP + α1AG + HP) at CGA of 30 Weeks

Because the APRs at CGA of 30 weeks were the earliest predictors of ROP occurrence, progression, and treatment, the APRs at this time point were compared to the other ROP predictive models: GA + BW, ROP score, and CHOP model (Table 4).

In Category 1, the discriminant probability of the APRs was 66%. When analyzed using the other three predictive models, however, the discriminant probability was 76% for all three models. When the APRs were incorporated into the three predictive models, the discriminant probability increased by 1% in the GA + BW and CHOP models.

In Category 2, the discriminant probability of the APRs was 67%; this was higher than all other predictive models, which had discriminant probabilities of 64–66%. The APRs were rated as the highest determinants of whether children would require treatment. When the APRs were incorporated into the three predictive models, the discriminant probability increased by 4–5% in each of the other models. The highest discriminant probability (71%) was achieved by GA + BW + APRs (*p* = 0.0007).

In Category 3, the discriminant probability of the APRs was 75%. Similarly, when the APRs were incorporated into the three predictive models, the discriminant probability increased by 3–5%.

The following discriminant probability formulas were utilized to determine both the onset and the severity of ROP requiring treatment.

### 3.6. Discriminant Probability Formula for Each Case (Table 5)

The discriminant probability formula for ROP occurrence is as follows.

**Z1:** Discriminant probability formula for ROP occurrence = 8.483 − 0.1699 × GA − 0.003125 × BW − 0.071678 × CRP + 0.003203 × α1AG + 0.0141 × HP (Z1 ≥ 0 indicates ROP occurrence, Z1 < 0 indicates no occurrence);

8.483 is the constant term. The discriminant coefficients for each predictor are: −1.699 for GA, −0.003125 for BW, −0.071678 for CRP, +0.003203 for α1AG, +0.0141 for HP. A positive coefficient indicates that an increase in the corresponding predictor raises the probability of ROP occurrence, while a negative coefficient suggests a protective effect.

The discriminant score (**Z1**) is interpreted as follows: **Z1** ≥ **0** indicates ROP occurrence. **Z1** < **0** indicates no ROP occurrence. This model highlights the balance between risk-enhancing and protective factors in predicting ROP. **Z2** and **Z3** are derived using a similar formula to **Z1**.

**Z2:** Discriminant probability formula that treatment is necessary in cases ROP develops = 8.401 − 0.4414 × GA + 0.00241 × BW − 0.2497 × CRP + 0.03031 × α1AG + 0.004085 × HP (Z2 ≥ 0 indicates no treatment, Z2 < 0 indicates treatment);

**Z3:** Discriminant probability formula that treatment is necessary in all cases = 4.486 − 0.21 × GA − 0.0005016 × BW − 0.2219 × CRP + 0.03259 × α1AG + 0.02033 × HP (Z3 ≥ 0 indicates no treatment, Z3 < 0 indicates treatment).

**Table 5:** Discriminant coefficients for determining each discriminant probability formula.

	Category 1		Category 2		Category 3	
	No ROP vs. Mild ROP + Treated ROP		Mild ROP vs. Treated ROP		No ROP + Mild ROP vs. Treated ROP	
Constant	8.843		8.401		4.486	
	Discriminant Coefficient	*p*-Value	Discriminant Coefficient	*p*-Value	Discriminant Coefficient (%)	*p*-Value
gestation age	−1.699	2.2 × 10^−3^	−0.4414	7.7 × 10^−3^	−0.2096	0.019
birth weight	−0.003125	8.1 × 10^−8^	0.00241	0.096	−0.0005016	0.62
CRP (CGA 30 W)	−0.07168	0.34	−0.2497	0.041	−0.2219	0.066
α1AG (CGA 30 W)	0.003203	0.69	0.03031	0.017	0.03259	0.012
HP (CGA 30 W)	0.0141	0.064	0.004085	0.69	0.02033	0.1

Discriminant analysis using Hotelling’s T-squared Test.

### 3.7. ROC Analysis for Category 1, 2, 3

Sensitivity, specificity, accuracy, and area under the ROC curve were good in all categories. The three ROC curve figures are provided in the Supplementary File (Table 6).

## 4. Discussion

ROP presents a significant challenge because its treatment in the first 80 days of life can affect the lifelong quality of a child’s vision [24]. Although ROP often heals spontaneously, prompt treatment is necessary when the condition reaches its limits [25]. Therefore, there are ongoing searches for predictors of the onset and progression of active ROP to detect children requiring treatment. Although gestation and birth weight are important predictors of development, it is important to use other biomarkers, such as genes and biological response.

Several recent reports have focused on the use of cytokines to predict the progression and severity of ROP. Cytokines are very effective predictors, but cytokines have short half-lives, and frequent testing is necessary in practice to assess the severity of ROP. Moreover, discrepancies exist. For example, Silveira et al. [9] reported high interleukin 6 (IL-6) levels at 72 h of age, whereas Cheng et al. [10] reported low IL-6 levels. Holm et al. [11] found no IL-6 abnormalities in early (0–14 days) samples, but high IL-6 levels in late (14–28 days) samples. Sood et al. [12] also reported conflicting results, with normal IL-6 levels in patients without ROP and patients with treated ROP, but elevated IL-6 levels in those with mild ROP.

These discrepancies highlight the challenge correlating individual cytokines with ROP onset and severity, because cytokines have multiple opposing effects, including anti-inflammatory and proinflammatory effects as well as anti-angiogenic and angiogenic effects depending on the target tissue, dosage, and timing of measurement [9,10,11,12].

Holms et al. [11] demonstrated that the concentrations of inflammatory mediators were more closely related to ROP severity at 14–28 days of age than at 14 days of life in premature infants with a GA less than 28 weeks. This means that the timing of elevated inflammatory mediators is involved in the development or worsening of ROP in premature patients.

Classically, the pathogenesis of ROP is divided into two phases [1,4,8]. The first phase is characterized by the cessation of retinal vessel formation and maturation and the second by the formation and growth of new abnormal vessels due to the generation of cytokines, such as VEGF. Some premature infants may develop Phase 1, but not Phase 2. The large ELGAN study found that late bacteremia is associated with an increased risk of severe ROP [6], and it can be assumed that patients who develop Phase 2 after Phase 1 have elevated systemic inflammatory mediators before Phase 2. This is why we focused on APR.

To use inflammatory markers as predictors of ROP severity, optimal timing of measurement is necessary. Since the second phase of ROP begins from CGA 30 W to 32 W [9], the APRs that we examined at CGA 30 W to 32 W were between the first and second phases, and we believe that we chose the appropriate time for testing.

In this study, the highest values in the first 72 h of life were not predictors. The highest value of APR at up to CGA 32 W was a significant biomarker for both onset and treatment prediction. Furthermore, the highest value of APR at up to CGA 30 W was still a significant biomarker for predicting both disease onset and treatment. In terms of predicting treatment, Categories 2 and 3 had significantly higher discrimination probabilities over Category 1 (Table 4), suggesting that APR is a better predictor for determining the likelihood of having treatment than the onset of ROP.

Alpha1AG, the best predictive marker in this study, is a stronger early predictor of mortality in neonatal sepsis than CRP [17,18,26]. It is regulated by IL-1 and hepatocyte growth factor (HGF), and in vitro experiments have demonstrated that HGF inhibits α1AG synthesis [27]. Many cytokine concentrations are higher in children with ROP than in controls [10], while monocyte chemotactic protein-1 and HGF levels are lower [28].

In addition, a report on cytokines in the subretinal fluid of patients with ROP with retinal detachment found that HGF was low and VEGF was high up to stage 4, when the detachment began, and that HGF and VEGF were at the same concentration at stage 5, when the detachment was extensive [29]. This is thought to reflect the pathology of ROP, where HGF is low when the active stage is worsening, and HGF may be overproduced and rises as the disease progresses to stage 5 and the healthy retina is gone. In any case, since HGF is low in subretinal fluid and serum when ROP is active and the patient’s condition is poor, it is inferred that high α1AG, which is not suppressed by HGF, indicates that the child is in an active stage of ROP that is likely to worsen and progress to requiring therapy. Since α1AG can be detected for 120–144 h after its expression [30], measuring it once a week up to CGA 30 W should provide valid data.

If APR results can be obtained before CGA 30 W, when the fundus, which has become cloudy due to vitreous opacity and lens vascular membrane dilation, becomes easier to see, it is possible to predict whether the patient requires ROP therapy and ophthalmologists and plan a careful response by performing a fundus examination. If the APR is negative, the number of ophthalmologic examinations could be reduced, preserving the child’s good physical condition and saving ophthalmologists and pediatricians time.

This study also included children with older gestational ages and higher birth weights. Today, a significant number of children lose their sight due to ROP in many countries. It is hoped that this examination will be adopted in such countries and used to initiate the treatment of ROP. Frequent testing all the three APR is not cost-effective. Therefore, we recommend α1AG, since it is the most promising APR, instead of CRP once a week.

### Strength and Limitations

The results were not obtained from samples used exclusively for research, but from serum samples in routine medical examination for premature infants. Accurate multiple parameters were recorded without data missing in a single center. On the other hand, data from a single institution has the weakness of bias and small sample data. Ultimately, prospective studies involving more patients should be conducted.

## Figures and Tables

**Table 1:** Characteristics of the infants examined in this study.

	Total	No ROP	Mild ROP	Treated ROP	*p*-Value
Predictive Factors	(*n* = 290)	(*n* = 155)	(*n* = 112)	(*n* = 23)	
Baseline					
gestational age, mean (SD), week	29.3 ± 3.5	30.9 ± 3.5	27.9 ± 2.5	25.9 ± 2.3	<0.0001 *
birthweight, mean (SD), g	1227 ± 338	1300 ± 266	951 ± 290	817 ± 329	<0.0001 *
male, no. (%)	141 (49)	72 (47)	52 (46)	17 (74)	0.041 ^†^
Pulmonary function: No. (%)					
continuous positive airway pressure	210 (76)	91 (63)	96 (87)	23 (100)	<0.0001 ^†^
use history of oxygen	145 (50)	51 (33)	77 (69)	17 (74)	<0.0001 ^†^
respiratory distress syndrome	140 (48)	57 (37)	67 (60)	16 (70)	<0.0001 ^†^
Circulatory function: No. (%)					
patent ductus arteriosus ligation	136 (47)	50 (32)	72 (64)	14 (61)	<0.0001 ^†^
blood transfusion	69 (24)	11 (7)	41 (37)	17 (74)	<0.0001 ^†^
Central nervous systems: No. (%)					
intraventricular hemorrhage	26 (9.0)	7 (4.5)	15 (13)	4 (17)	0.015 ^†^
periventricular leukomalacia	14 (4.8)	8 (5.2)	4 (3.6)	2 (8.7)	0.56 ^†^
Growth: Weight gain: mean (SD), g					
from birth till 6 weeks	564 (292)	691 (271)	442 (247)	307 (204)	<0.0001 *
rate from birth till 6 weeks/day	13 (7.0)	16 (6.5)	11 (5.9)	7.3 (4.9)	<0.0001 *
from 8 till 15 days	71 (89)	90 (93)	48 (80)	50 (75)	<0.0001 *
rate 8 till 15 days/day	11 (12)	13 (12)	7.0 (12)	7.1 (11)	<0.001 *
Acute Phase Protein: Mean (SD), g/dL					
up to 72 H CRP	0.4 (1.3)	0.4 (1.8)	0.3 (0.5)	0.3 (0.51)	0.083 *
α1AG	20 (15)	19 (15)	20 (17)	24 (14)	0.31 *
HP	11 (15)	9 (7)	13 (20)	15 (285)	0.057 *
CGA 30 W CRP	0.8 (2.4)	0.5 (1.8)	1.2 (2.6)	1.6 (3.8)	0.017 *
α1AG	32 (28)	23 (19)	39 (31)	62 (32)	<0.0001 *
HP	18 (27)	9.9 (11)	23 (34)	42 (40)	<0.0001 *
CGA 33 W CRP	1.1 (3.2)	0.5 (1.8)	1.5 (3.2)	3.1 (6.9)	<0.001 *
α1AG	39 (33)	27 (21)	47 (35)	78 (45)	<0.0001 *
HP	19 (28)	11 (12)	25 (34)	47 (44)	<0.0001 *
CGA 35 W CRP	1.1 (3.2)	0.5 (1.8)	1.5 (3.2)	3.1 (6.9)	<0.001 *
α1AG	39 (33)	27 (22)	47 (35)	77 (45)	<0.0001 *
HP	19 (28)	11 (12)	25 (34)	47 (44)	<0.0001 *

Linear discriminant analysis of the three ROP classification groups showed that α1AG and HP at CGA of 30, 33, and 35 weeks were highly significant (*p* < 0.0001). Abbreviations: ROP—retinopathy of prematurity, CRP—C-reactive protein, α1AG—alpha 1 acid glycoprotein, HP—haptoglobin, up to 72 H—the highest value during the first 72 h after birth, CGA 30 W, CGA 33 W, CGA 35 W—the highest value between birth and each CGA weeks, CGA—corrected gestational age. ^†^ chi-squared test, * one-way layout analysis of variance.

**Table 2:** Predictability of each acute phase reactants in Categories 1, 2, and 3.

		Category 1		Category 2		Category 3	
		No ROP vs. Mild ROP + Treated ROP		Mild ROP vs. Treated ROP		No ROP + Mild ROP vs. Treated ROP	
Different Times	APR	Discriminant Coefficient	*p*-Value	Discriminant Coefficient	*p*-Value	Discriminant Coefficient	*p*-Value
up to 72 H	CRP	−0.098	0.3	−0.6	0.37	−0.12	0.5
	α1AG	0.002	0.81	0.03	0.19	0.017	0.3
	HP	0.019	0.037 *	0.005	0.75	0.014	0.39
CGA 30 W	CRP	−0.059	0.36	−0.23	0.047 *	−0.22	0.06
	α1AG	0.024	0.0002 ***	0.03	0.006 **	0.04	0.0003 ***
	HP	0.013	0.06	0.01	0.31	0.23	0.05
CGA 33 W	CRP	−0.062	0.23	−0.094	0.27	−0.07	0.45
	α1AG	0.025	<0.0001 ***	0.025	0.01 *	0.04	0.0003 ***
	HP	0.012	0.04 *	0.007	0.39	0.02	0.059
CGA 35 W	CRP	−0.065	0.21	−0.094	0.27	−0.07	0.44
	α1AG	0.026	<0.0001 ***	0.025	0.01 *	0.04	0.0003 ***
	HP	0.012	0.046 *	0.008	0.36	0.02	0.05

To examine the nature of the three inflammatory proteins, they were analyzed at different growth times and compared between the different two groups. A multivariate analysis was used in which the discrimination coefficients were adjusted among the three items. Abbreviations: ROP—retinopathy of prematurity, CRP—C-reactive protein, α1AG—alpha-1 acid glycoprotein, HP—haptoglobin, up to 72 H—the highest value during the first 72 h after birth, CGA 30 W, CGA 33 W, CGA 35 W—the highest value between birth and each CGA weeks, CGA—corrected gestational age. Statistical significance is denoted by asterisks: * *p* < 0.05; ** *p* < 0.01; *** *p* < 0.001.

**Table 3:** Multivariate-adjusted analysis for all acute phase reactants (CRP + α1AG + HP) in Categories 1, 2, and 3.

		Category 1			Category 2			Category 3		
		No ROP vs. Mild ROP + Treated ROP			Mild ROP vs. Treated ROP			No ROP + Mild ROP vs. Treated ROP		
Difference Times	APR	Discriminant Probability (%)	Discriminant Coefficient	*p*-Value	Discriminant Probability (%)	Discriminant Coefficient	*p*-Value	Discriminant Probability (%)	Discriminant Coefficient	*p*-Value
up to 72 H	CRP + α1AG + HP	56	−0.21	0.11	57	−0.42	0.57	58	−0.51	0.37
CGA 30 W	CRP + α1AG + HP	66	−0.97	<0.0001 ***	67	−1.52	<0.0001 ***	75	−2.34	<0.0001 ***
CGA 33 W	CRP + α1AG + HP	67	−1.15	<0.0001 ***	67	−1.62	<0.0001 ***	76	−2.59	<0.0001***
CGA 35 W	CRP + α1AG + HP	67	−1.16	<0.0001 ***	67	−1.61	<0.0001 ***	76	−2.59	<0.0001 ***

The three inflammatory proteins were summed and adjusted to compare the discrimination probabilities and coefficients between the two different groups at different growth times. Adjusted using multivariate analysis (Mahalanobis square distance). Abbreviations: ROP—retinopathy of prematurity, CRP—C-reactive protein, α1AG—alpha-1 acid glycoprotein, HP—haptoglobin, up to 72 H—the highest value during the first 72 h after birth, CGA 30 W, CGA 33 W, CGA 35 W—the highest value between birth and each CGA weeks. CGA—corrected gestational age. Statistical significance: *** *p* < 0.001.

**Table 4:** Predictability of GA + BW, ROP score, and CHOP models and impacts of all acute phase reactants (CRP + α1AG + HP) at CGA of 30 weeks.

	Category 1				Category 2				Category 3			
	No ROP vs. Mild ROP + Treated ROP				Mild ROP vs. Treated ROP				No ROP + Mild ROP vs. Treated ROP			
Predictive Factors	Discriminant Probability (%)		Discriminant Coefficient	*p*-Value	Discriminant Probability (%)		Discriminant Coefficient	*p*-Value	Discriminant Probability (%)		Discriminant Coefficient	*p*-Value
CRP + α1AG + HP (CGA 30 W)	66		−0.966563	<0.0001 ***	67		−1.523	<0.0001 ***	75		−2.33792	<0.0001 ***
GA + BW	76	] 1%	9.42288	<0.0001 ***	66	] 5%	10.9711	0.0015 **	73	] 5%	1.44807	<0.0001 ***
CRP + α1AG + HP (CGA 30 W) + GA + BW	77		8.483	<0.0001 ***	71		8.40139	0.0007 ***	78		4.48555	<0.0001 ***
ROP score	76		−7.84552	<0.0001 ***	65	] 4%	−4.84456	0.0011 **	75	] 4%	−7.17012	<0.0001 ***
CRP + α1AG + HP (CGA 30 W) + ROP score	76		−7.63806	<0.0001 ***	69		−4.4066	0.0015 **	79		−6.70968	<0.0001 ***
CHOP model	76	] 1%	3.00313	<0.0001 ***	64	] 4%	0.710825	0.0029 **	75	] 3%	1.72822	<0.0001 ***
CRP + α1AG + HP (CGA 30 W) + CHOP model	77		2.67486	<0.0001 ***	68		−0.74102	0.0028 **	78		−0.318112	<0.0001 ***

The growth time of the three inflammatory protein prediction models was set at 30 W and compared to other prediction models, discrimination probabilities, and discrimination coefficients between the two different groups. The predictors of this study were loaded onto the other prediction models, showing improvement in discrimination probability. Multivariate analysis was used to adjust for Mahalanobis square distance. Based on APR analysis of CGA 30 W, GA + BW, ROP score, and CHOP model were combined for analysis and comparison. Abbreviations: ROP—retinopathy of prematurity, GA—gestational age, BW—birthweight, CRP—C-reactive protein, α1AG—alpha-1 acid glycoprotein, HP—haptoglobin. CGA 30 W—the highest value between birth and 30 CGA weeks. CGA—corrected gestational age. Statistical significance: ** *p* < 0.01; *** *p* < 0.001. The degree of improvement between each predictor is indicated by the symbol ‘]’.

**Table 6:** ROC analysis was performed with all variables: gestation age, birth weight, CRP (CGA 30 W), α1AG (CGA 30 W), and HP (CGA 30 W).

	Category 1		Category 2		Category 3	
Variable: Gestation Age, Birth Weigh CRP (CGA 30 W), α1AG (CGA 30 W), HP (CGA 30 W)	No ROP vs. Mild ROP + Treated ROP		Mild ROP vs. Treated ROP		No ROP + Mild ROP vs. Treated ROP	
Sensitivity (95% Confidence Interval)	0.7185	0.634697–0.792489	0.7391	0.515948–0.897714	0.6957	0.470808–0.867897
Specificity	0.8129	0.742479–0.870973	0.7232	0.63069–0.803566	0.8314	0.781059–0.874331
Accuracy	0.7690	0.716111–0.816231	0.7260	0.642543–0.799119	0.8207	0.771601–0.863087
Area under the ROC curve	0.8448	0.799727–0.889831	0.8032	0.721111–0.885255	0.8640	0.801212–0.926845

## Data Availability

The data that support the findings of this study are available from Nagoya City University West Medical Center but restrictions apply to the availability of these data, which were used under license for the current study, and so are not publicly available. Data are, however, available from the corresponding author upon reasonable request and with permission of Nagoya City University West Medical Center.

## Supplementary Materials

The following supporting information can be downloaded at: https://www.mdpi.com/article/10.3390/diagnostics15050571/s1, ROC Analysis for Category 1, 2, 3.
