# Supplementary material for: The Role of Acute Phase Reactants, Including α1-Acid Glycoprotein, in Predicting Onset and Severity of Retinopathy of Prematurity
Source: Diagnostics (Basel). 2025 Feb 27;15(5):571. doi: 10.3390/diagnostics15050571 (PMC11898616; doi:10.3390/diagnostics15050571)
Supplement: Supplementary file 1 [file diagnostics-15-00571-s001.zip › diagnostics-3465220-supplementary.pptx]

## Slide 1
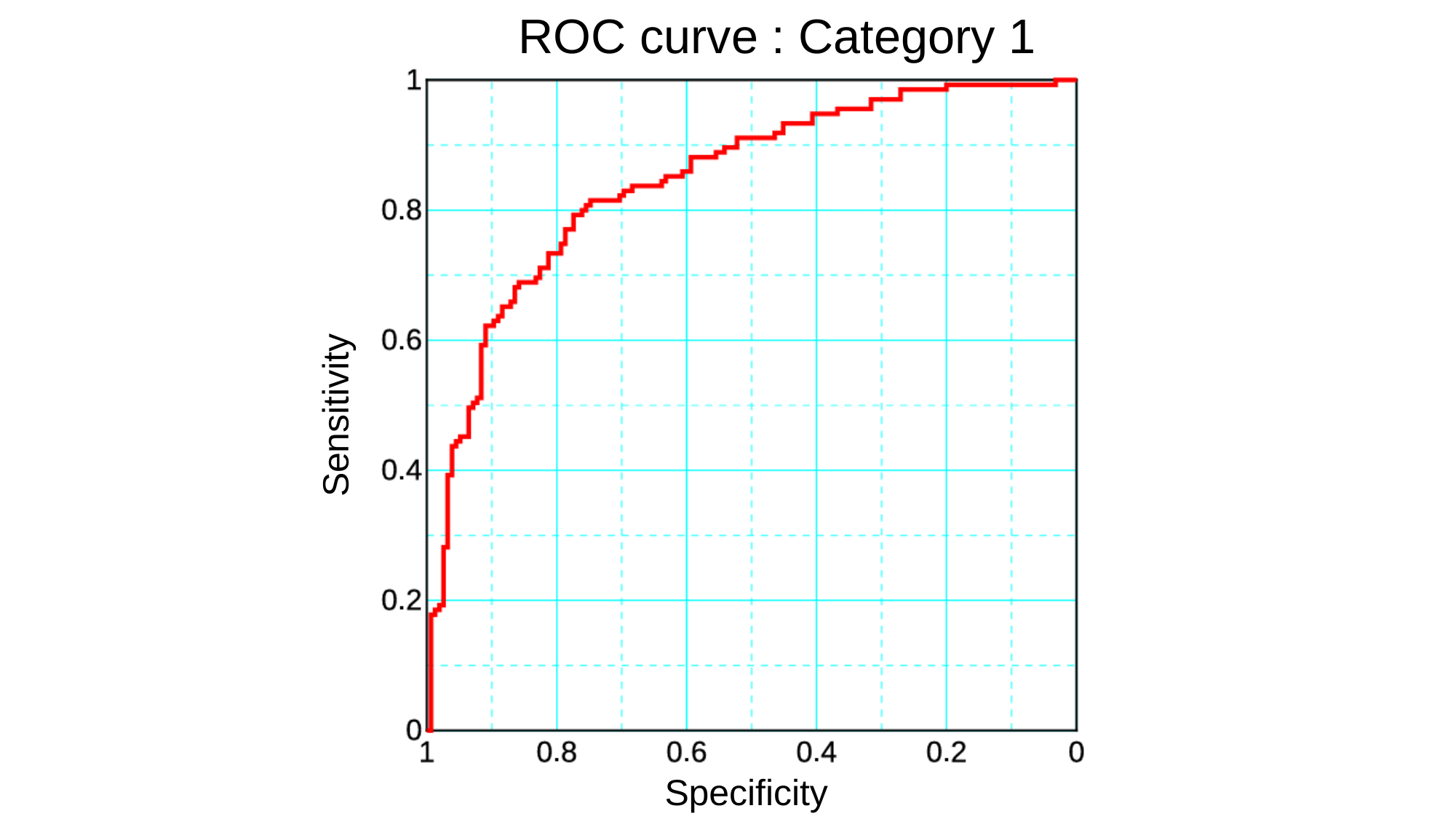

ROC curve : Category 1
Sensitivity
Specificity

## Slide 2
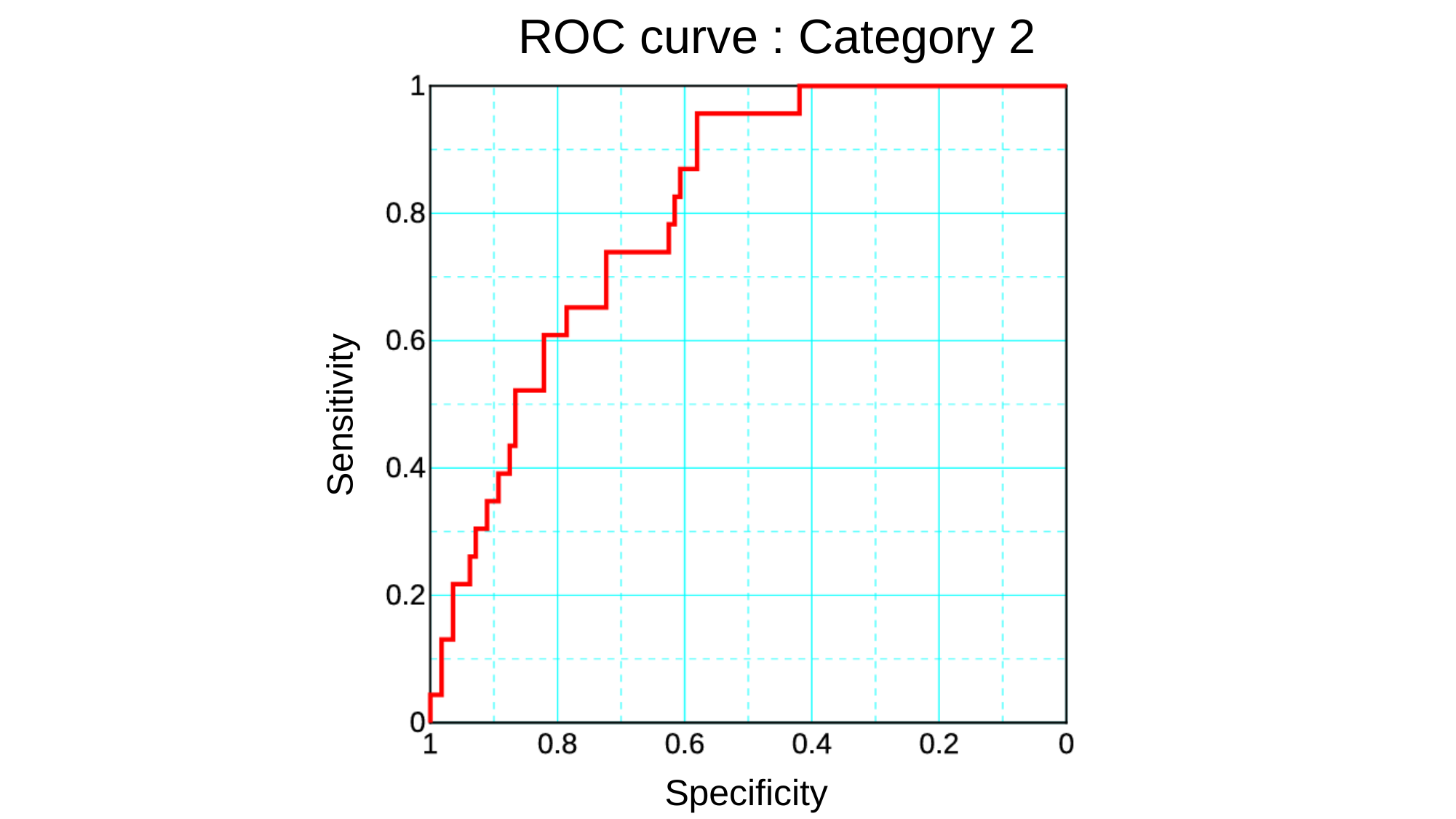

ROC curve : Category 2
Sensitivity
Specificity

## Slide 3
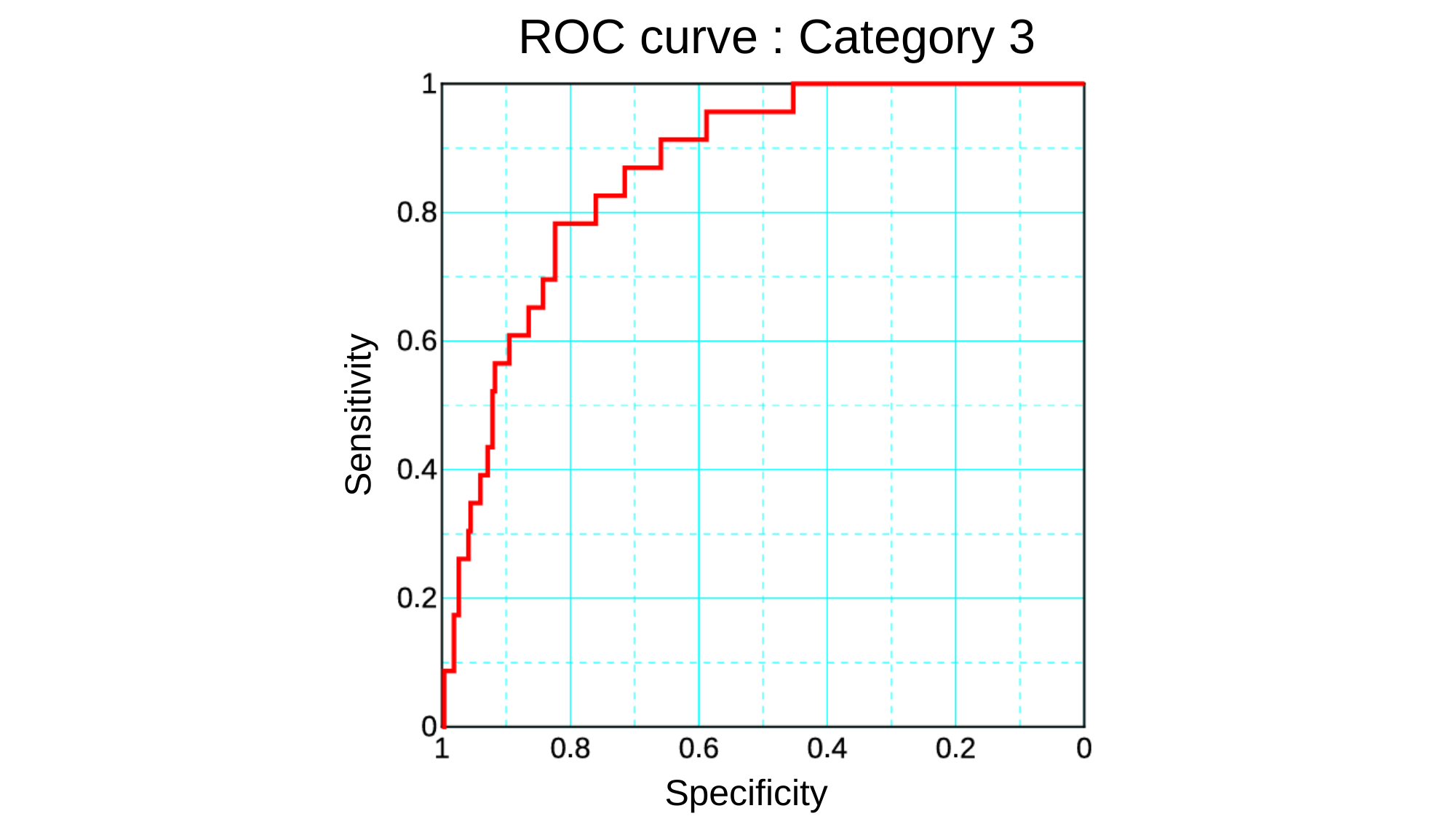

ROC curve : Category 3
Sensitivity
Specificity
